# Supplementary material for: Imaging the fate of histone Cse4 reveals de novo replacement in S phase and subsequent stable residence at centromeres
Source: eLife. 2014 May 20;3:e02203. doi: 10.7554/eLife.02203 (PMC4067749; doi:10.7554/eLife.02203)
Supplement: Supplementary file 1. — Batch FITS converter macro for ImageJ. This macro converts 16-bit TIFF files from a selected folder into FITS files (re-assigned into 32-bit floating-point space) and saves them in a destination folder. The content of the file should be saved as ‘Batch FITS Converter.txt’ into Macro folder of ImageJ. DOI: http://dx.doi.org/10.7554/eLife.02203.024 [file elife02203s001.docx]

// Batch Convert

//

// This macro converts all the files in a folder to FITS

// format. X /Y axes are properly oriented

// Three dialog boxes are displayed. Select the source

// folder in the first, the FITS in the second and the destination

// folder in the third.

dir1 = getDirectory("Choose Source Directory ");

format = getFormat();

dir2 = getDirectory("Choose Destination Directory ");

list = getFileList(dir1);

setBatchMode(true);

for (i=0; i<list.length; i++) {

showProgress(i+1, list.length);

open(dir1+list[i]);

run("Flip Vertically");

run("32-bit");

saveAs(format, dir2+list[i]);

close();

}

function getFormat() {

formats = newArray("FITS");

Dialog.create("Batch Convert");

Dialog.addChoice("Convert to: ", formats, "TIFF");

Dialog.show();

return Dialog.getChoice();

}
